# Identification of a *Plasmodium falciparum* Phospholipid Transfer Protein* [Fn FN2]

**DOI:** 10.1074/jbc.M113.474189

**Published:** 2013-09-16

**Authors:** Christiaan van Ooij, Chrislaine Withers-Martinez, Alessa Ringel, Shamshad Cockcroft, Kasturi Haldar, Michael J. Blackman

**Affiliations:** From the ‡ Division of Parasitology, MRC National Institute for Medical Research, Mill Hill, London NW7 1AA, United Kingdom,; the § Department of Neuroscience, Physiology and Pharmacology, University College London, London WC1E 6JJ, United Kingdom, and; the ¶ Center for Rare and Neglected Diseases and Department of Biological Sciences, University of Notre Dame, Notre Dame, Indiana 46556

**Keywords:** Lipid Transport, Malaria, Parasitology, Phospholipid, Plasmodium, Phospholipid Transfer

## Abstract

Infection of erythrocytes by the human malaria parasite *Plasmodium falciparum* results in dramatic modifications to the host cell, including changes to its antigenic and transport properties and the *de novo* formation of membranous compartments within the erythrocyte cytosol. These parasite-induced structures are implicated in the transport of nutrients, metabolic products, and parasite proteins, as well as in parasite virulence. However, very few of the parasite effector proteins that underlie remodeling of the host erythrocyte are functionally characterized. Using bioinformatic examination and modeling, we have found that the exported *P. falciparum* protein PFA0210c belongs to the START domain family, members of which mediate transfer of phospholipids, ceramide, or fatty acids between membranes. *In vitro* phospholipid transfer assays using recombinant PFA0210 confirmed that it can transfer phosphatidylcholine, phosphatidylinositol, phosphatidylethanolamine, and sphingomyelin between phospholipid vesicles. Furthermore, assays using HL60 cells containing radiolabeled phospholipids indicated that orthologs of PFA0210c can also transfer phosphatidylcholine, phosphatidylinositol, and phosphatidylethanolamine. Biochemical and immunochemical analysis showed that PFA0210c associates with membranes in infected erythrocytes at mature stages of intracellular parasite growth. Localization studies in live parasites revealed that the protein is present in the parasitophorous vacuole during growth and is later recruited to organelles in the parasite. Together these data suggest that PFA0210c plays a role in the formation of the membranous structures and nutrient phospholipid transfer in the malaria-parasitized erythrocyte.

## Teaser

**Background:** PFA0210c is an exported *Plasmodium falciparum* protein.

**Results:** PFA0210c is a phospholipid transfer protein with similarities to proteins of the family of START domain-containing proteins and can transfer various phospholipids between membranes *in vitro*.

**Conclusion:** PFA0210c and its orthologs are phospholipid transfer proteins with a broad specificity.

**Significance:** Identification of the function of conserved *Plasmodium* proteins allows deeper insight into the basis of pathogenesis.

## Introduction

*Plasmodium falciparum* is the causative agent of malaria, a disease that claims nearly one million lives each year (1, 2). All symptoms of malaria result from infection of erythrocytes by the parasite, so investigating interactions between the parasite and its host cell is of great importance to understanding of the disease. The parasite actively invades the erythrocyte and comes to rest within a membrane-bound parasitophorous vacuole (PV).^3^ The intracellular parasite develops into a trophozoite, which replicates asexually by a process called schizogony, gradually expanding to fill an increasing proportion of the host cell volume. Eventually, at ∼48 h post-invasion, the schizont undergoes segmentation to produce 16–32 progeny merozoites that are released upon rupture of the infected cell to repeat the cycle.

The parasitized erythrocyte undergoes a range of dramatic structural changes during parasite growth, including alterations to its deformability, membrane permeability, and cytoadhesive properties (3). Throughout its intracellular life cycle the parasite remains surrounded by the PV membrane (PVM), which therefore has to expand to accommodate the growing parasite. The parasite also induces the formation of additional membranous compartments within the cytosol of the infected erythrocyte beyond the PV, including Maurer clefts, which are important for the assembly of the surface-exposed knobs (4) that alter the adhesive properties of the erythrocyte, and the tubovesicular network, which plays an important role in nutrient import (5). Furthermore, two different types of mobile vesicles have been identified in infected erythrocytes; the highly mobile vesicles (6) and the J-dots (7). These structures may also have a role in the transport of parasite proteins throughout the erythrocyte. However, no parasite protein that might play a role in assembling the PVM and other induced membrane structures in the parasite-infected erythrocyte has been identified.

A large set of parasite proteins is known to be exported beyond the parasite plasma membrane and the PVM into the host erythrocyte (8). Identification of these proteins was aided by the discovery of a distinct export motif, the HT (or PE*X*EL) motif (9, 10), which directs many of the exported proteins into the host cell. Based on this motif it has been predicted that between 422 (11) and 455 (12) proteins are exported. The upper figure may be a conservative estimate as it has recently been recognized that several exported proteins do not contain the HT/PE*X*EL motif (13) but may be exported through the same common pathway (14, 15). Hence the total number of exported parasite proteins likely exceeds these estimates.

Exported *P. falciparum* proteins may be divided into three categories. The first comprises species-specific proteins, which likely have roles specific for *P. falciparum* and probably mediate specific aspects of its pathogenicity. The best studied of these is PfEMP1, the main component of the knob structures at the surface of the parasitized erythrocyte that are required for vascular sequestration. The second category comprises host-specific exported proteins, which are found only in *Plasmodium* species that infect humans and likely play roles in the interaction with the host. A third category comprises several exported proteins that are found in all sequenced *Plasmodium* species (11, 12). These likely perform important, conserved functions in the interaction of the parasite with the host cell and may represent the basic machinery that allows the parasite to survive inside the erythrocyte. However, the absence of obvious sequence similarity between exported malarial proteins and proteins in the databases means that the biochemical function of most of the exported parasite proteins is unknown.

Here we describe an investigation of the *P. falciparum* protein PFA0210c (current PlasmoDB ID PF3D7_0104200), previously identified by us (9) as one of the subset of exported proteins conserved in all *Plasmodium* species examined (11). Using bioinformatic and biochemical approaches, we demonstrate that PFA0210c is a member of the family of steroidogenic acute regulatory protein-related lipid transfer (START) domain-containing phospholipid transfer proteins. We show that PFA0210c is expressed during the asexual intraerythrocytic life cycle, associates with membranes, and has the capacity to transfer phospholipids between phospholipid vesicles.

## EXPERIMENTAL PROCEDURES

### 

#### 

##### Cloning and Expression Constructs

Gene fragments of *PFA0210c*, *PKH*_*020910*, and *PCHAS*_*020730* were amplified from genomic DNA by PCR using the primers listed in supplemental Table S1. The resulting fragments were digested with EcoRI and XhoI and cloned into pMAL c2x (New England Biolabs) that had been digested with EcoRI and SalI. The resulting plasmids encode a fusion protein with maltose-binding protein (MBP) at the N terminus and a C-terminal hexahistidine (His_6_) tag. Point mutations were produced by overlapping PCR using the primers listed in supplemental Table S1. The PCR fragments were fused in a second PCR step using both amplicons as templates in conjunction with external primers (supplemental Table S1). The resulting DNA was further amplified with primers CVO022 and CVO023 and cloned into pMAL c2x as described above. An additional 5′ sequence was added by cloning the EcoRV-BsrGI fragment from pBLD368 into the resulting plasmids.

##### Protein Purification

Recombinant proteins were purified as MBP-His_6_ fusions from *Escherichia coli* BL21. Protein production was induced by addition of isopropyl β-d-1-thiogalactopyranoside (0.5 mm) to bacterial cultures, which were then grown overnight at 18 °C. The cultures were harvested by centrifugation, followed by suspension in high salt MBP buffer (20 mm Tris-HCl, pH 7.4, 0.5 m NaCl) containing protease inhibitors (Complete EDTA-free Mixture, Roche Applied Science) and lysed with a Cell Disruptor (Constant Cell Disruption System). The lysate was sonicated for three cycles of 30 s, setting 4, 50% duty cycle with a microtip (Vibracell, Sonics and Materials, Inc.) and subsequently centrifuged twice in an Oakridge tube at 9,000 × *g* for 30 min in a JA25.5 rotor. The supernatant was then transferred to a 50-ml conical tube containing 0.5–1.0 ml of Ni^2+^-NTA resin (Qiagen), and mixed at 4 °C for 2 h. The resin was transferred to a 1.5 × 12-cm chromatography column (Bio-Rad), washed with ∼100 column volumes of high salt MBP buffer containing 25 mm imidazole, then bound protein was eluted with 10 ml of high salt MBP buffer containing 250 mm imidazole. For the time course and titration experiments, the Ni^2+^-NTA eluate was polished by gel filtration chromatography on a HiLoad 26/60 Superdex 200 prep-grade column equilibrated in standard assay buffer (10 mm HEPES-Na^+^, 1 mm EDTA-Na^+^, 50 mm NaCl, pH 7.4). Peak protein fractions were identified by SDS-PAGE and Coomassie staining, and concentrated using a 30-kDa molecular mass cut-off Vivaspin 15 concentrator (Sartorium Stedim Biotech). The protein concentration was determined using a Nanodrop 1000 (Thermo Scientific) and the concentrated protein was stored at 4 °C for up to 2 weeks without a noticeable decrease in activity. To purify recombinant proteins used for the remaining transfer assays, the Ni^2+^-NTA eluate was added to ∼1 ml of amylose resin (New England Biolabs) and incubated at 4 °C overnight with agitation. The resin was poured into a 1.5 × 12-cm chromatography column (Bio-Rad), washed with ∼100 column volumes of MBP buffer (20 mm Tris-HCl, pH 7.4, 0.2 m NaCl), and bound protein was eluted with 10 ml of MBP buffer containing 50 mm maltose. The eluate was dialyzed against standard assay buffer (10 mm HEPES-Na, 1 mm EDTA-Na, 50 mm NaCl, pH 7.4) using Slide-A-Lyzer cassettes (Thermo Scientific) with a molecular mass cut-off of 20,000 Da. The protein was subsequently concentrated and the concentration was established as described above. The proteins were divided into aliquots, snap frozen in a dry ice-ethanol bath, and stored at −80 °C. After thawing, the proteins were stored at 4 °C and used for up to 2 weeks.

MBP-LacZ was purified similar to MBP-PFA0210c-His_6_, except that the bacterial lysate, after centrifugation, was loaded directly onto amylose resin and the protein was dialyzed against PBS using Slide-A-Lyzer cassettes (Thermo Scientific). It was then concentrated and frozen as described for MBP-PFA0210c-His_6_. Rat phosphatidylinositol transfer protein α (PITP) was purified as described previously (16).

##### Secondary Structure Predictions and Structure-based Homology Determination

The predicted PFA0210c primary amino acid sequence was submitted to the Profile Based Fold Recognition prediction method pGenTHREADER (17) (UCL, Bioinformatics Group) to generate modeling alignments of PFA0210c with homologous or related proteins from the Protein Data Bank (PDB).

##### Antibodies and Monoclonal Antibody Production

Monoclonal antibodies 24C6.1F1 (a kind gift of Jean-Francois Dubremetz, University of Montpellier 2) and X509, reactive with *P. falciparum* proteins SERA5 and MSP1, respectively, have been described previously (18, 19). To detect the HA epitope on Western blots, mAb 3F10 was used. The anti-PFA0210c monoclonal antibody (mAb) NIMP.M11 (subclass IgG1 as determined by Ouchterlony assay) was produced using splenic B cells obtained from BALB/c mice immunized with recombinant protein MBP-PFA0210c-His_6_ purified as described above. The cells were fused with Sp2/0/Ag14 myeloma cells and cloned by limiting dilution using standard procedures, screening cell supernatants by dot blot against a glutathione *S*-transferase-PFA0210c fusion protein that had been purified from *E. coli* on glutathione-agarose (Sigma) as per the manufacturer's instructions. Undiluted hybridoma supernatant was used for Western blots.

##### Detection of PFA0210c during the Intraerythrocytic Life Cycle

To obtain parasite extract over the course of one intraerythrocytic cycle, six small flasks containing a low hematocrit (∼1%) infected at a high parasitemia (∼20–30%) with *P. falciparum* clone 3D7 in 10 ml of RPMI containing Albumax were set up. To ensure that the parasites were synchronized, the erythrocytes were infected with Percoll-purified schizonts for 1 h. After this period, residual schizonts were removed on a Percoll gradient and any remaining schizonts were lysed by treatment with 5% (w/v) sorbitol. At the indicated time points a culture was pelleted by centrifugation, and transferred to two microcentrifuge tubes, washed once with 900 μl of PBS and stored at −80 °C. For Western blot analysis, each sample was solubilized directly into 200 μl of 1.2 × SDS-PAGE loading buffer and fractionated on 10% SDS-polyacrylamide gels. Proteins were transferred to nitrocellulose (Hybond-C Extra, GE Healthcare) and detected using the anti-PFA0210c mAb NIMP.M11, an HRP-linked secondary anti-mouse antibody, and a chemiluminescence developing system (Immobilon Western Chemiluminuescent HRP substrate, Millipore).

##### Construction of P. falciparum Expression Plasmids and Parasite Lines

pBLD390 was produced by amplifying the 5′ and 3′ regions of *PFA0210c* by PCR with primer pairs CVO001 and CVO002 and CVO024 and CVO023, respectively. The His_6_-HA_3_-GFP module was amplified from a version of pHH1SERA5chimWT (20) in which the His_6_-HA_3_-GFP module was inserted using primers CVO003 and CVO004. The three fragments were joined in one PCR using primers CVO001 and CVO023. The resulting DNA fragment was cloned into pGEM-T according to the supplier's instructions and subsequently cloned into pBLD210 (11). pBLD397 was produced by amplifying PFA0210c and 1938 bases of its upstream flanking (promoter) region using primers CVO030 and CVO023. The resulting fragment was cloned into pGEM-T according to the supplier's instructions. The His_6_-HA_3_-GFP module was introduced into the resulting plasmid by transferring a BstZ17I fragment and a BstZ17I-XhoI fragment from pBLD390. The resulting fusion was subsequently cloned into pA289 (a kind gift of Andrew Osborne) from which the SalI-CelII fragment, containing the calmodulin promoter, was removed using SpeI and XhoI.

PfBLD390 was created by transfecting schizonts of *P. falciparum* strain 3D7 with pBLD390 according to the protocol described by Moon *et al.* (21). PfBLD397 was created by transfecting the *P. falciparum* 3D7 strain containing a genomic integrated attB cassette (22) with pBLD397 and pINT, which mediates expression of the Bxb1 integrase in *P. falciparum* (22). Transfectants were selected with WR99210 in the case of PfBLD390 and blasticidin in the case of PfBLD397.

##### Parasite Extract Production and Fractionation, Western Blotting, and Streptolysin O Treatment

Percoll-enriched schizonts that had been frozen at −80 °C were thawed directly into hypotonic lysis buffer (10 mm Tris-HCl, pH 8.0, 5 mm EDTA) containing protease inhibitors. The lysate was incubated on ice for 90 min with occasional agitation, then divided into two aliquots and centrifuged at ∼100,000 × *g* in a Beckman TL-100 centrifuge using a TLA100.3 rotor. The supernatants containing soluble extract proteins were retained and the pellets were washed twice with hypotonic lysis buffer. Pellets then were resuspended in 0.7 ml of high salt buffer (50 mm Tris-HCl, 5 mm EDTA, 500 mm NaCl) by trituration and incubated on ice for a further 30 min. The samples were centrifuged as before, the supernatants were transferred to a new tube (this represents the membrane-associated fraction), and the pellets were resuspended in carbonate buffer (0.1 m sodium carbonate, pH 11) by trituration. The samples were kept on ice and centrifuged as before. The supernatant was transferred to a new tube and the pellet was resuspended in 1× SDS-PAGE loading buffer. The samples were run on a 10% SDS-polyacrylamide gel and the proteins transferred to nitrocellulose (Hybond-C Extra, Amersham Biosciences). PFA0210c was detected using mAb NIMP.M11 and SERA5 was detected in parallel using the anti-SERA5 mAb 24C6.1F1. The anti-SERA5 blot was stripped with 5% acetic acid and reprobed with the anti-MSP1 mAb X509. Streptolysin O treatment of infected erythrocytes was performed as described (23).

##### Phospholipid Transfer Assay

Phospholipid transfer was measured as described previously (24), except that the volume was reduced to 100 μl and the final spin was repeated to reduce the carry-over of pelleted vesicles. Radioactive l-[α-dipalmitoyl-1-^14^C]phosphatidylcholine and choline-[*methyl*-^3^H]sphingomylein (egg) were obtained from PerkinElmer Life Sciences.

##### Transfer of Radiolabeled Lipids to Liposomes Using Permeabilized HL60 Cells as a Donor

Association of cellular lipids with the PITP proteins was analyzed using a modification of a previously published protocol (25, 26). In brief, HL60 cells (50 ml) were labeled with 1 μCi/ml of [^14^C]acetate in RPMI 1640 medium for 48 h. The cells were permeabilized with streptolysin O and the leaked cytosol removed by centrifugation. 200 μl of permeabilized cells (∼10^7^ cells) were incubated with 50 μg of recombinant protein or a control protein (100 μl) for 60 min at 37 °C in the presence of 2 mm Mg^2+^-ATP and 200 μl of liposomes (PC:PI, 98:2 mol/mol, 160 μg of total lipid). The reaction was quenched by the addition of 100 μl of ice-cold 0.2 m sodium acetate, 0.25 m sucrose (pH 5.0). The tubes were vortexed and left on ice for 10 min to aggregate the membranes. Following centrifugation at 12,000 × *g* for 10 min at 4 °C, 500 μl of supernatant from each reaction was transferred to microcentrifuge tubes and further centrifuged at 100,000 × *g* in a benchtop ULTRA centrifuge for 1 h to remove any cellular debris. At the end of the incubation, 400 μl of the supernatant was sampled and the lipids were extracted and resolved by analytical thin layer chromatography (TLC) developed in chloroform:methanol:acetic acid:water (75:45:3:1, by volume). The TLC plates were imaged between 2 and 3 days using Fuji phosphorimaging screens and data were quantified by densitometry using AIDA software. Results are expressed as a percentage of the total input count. Error bars indicate mean ± S.D.

##### Purification of ^14^C-Labeled Phosphatidylinositol and Phosphatidylethanolamine

^14^C-Labeled phosphatidylinositol and phosphatidylethanolamine were purified from HL60 cells grown in [^14^C]acetate for 72 h. Cells (200 ml) were cultured in RPMI supplemented with 10% FCS and 1 μC/ml of [^14^C]acetate. After 72 h, the cells were pelleted by centrifugation and the lipids were extracted as described previously (25). The lipids were separated on a Whatman Silica Gel 60 TLC plate using chloroform:methanol:acetic acid:water (75:45:3:1) as the solvent. The TLC plate was exposed to a FUJI BAS1000 phosphorimaging system for 16 h. Phosphatidylinositol and phosphatidylethanolamine were located using standards and the silica containing PI and PE were scraped into glass tubes and the lipids were extracted from the silica. The lipid extract was used without further purification.

##### Live Fluorescence Microscopy

To prepare parasites for fluorescence microscopy, parasites were synchronized by adding Percoll-enriched mature schizonts to fresh erythrocytes and allowed to undergo invasion for ∼1 h, after which unruptured schizonts were removed by centrifugation on a second Percoll gradient. A small aliquot of parasitized erythrocytes was transferred to a microcentrifuge tube at the indicated time, centrifuged briefly, and resuspended in PBS containing 1 μg/ml of Hoechst 33258. After 5 min the cells were washed once with PBS and subsequently resuspended in a small volume of PBS. A 3-μl aliquot was used for microscopy.

## RESULTS

### 

#### 

##### PFA0210c Shares Sequence and Structural Similarity with Phospholipid Transfer Proteins

**FIGURE 1.**
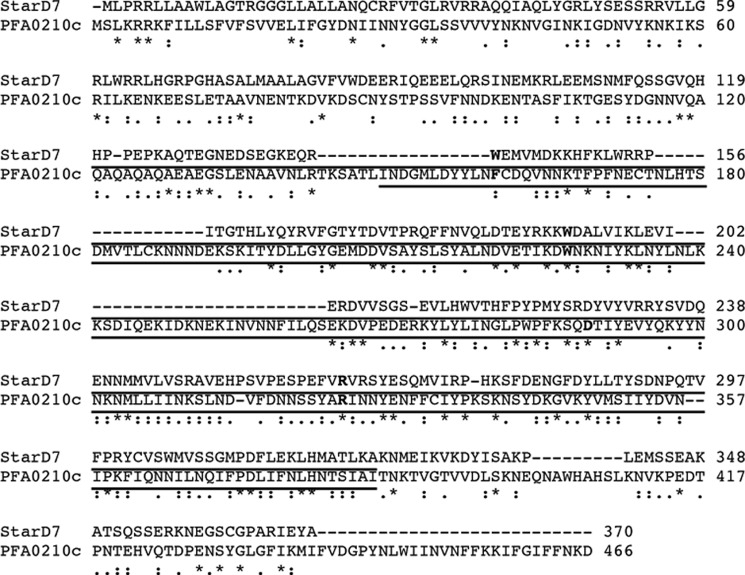
**PFA0210c exhibits similarity to START domain-containing proteins.** Alignment of PFA0210c and STARD7. Sequences of STARD7 and PFA0210c were aligned using the ClustalW program. Identical residues are indicated by *asterisks* (*), strongly similar residues by *colons* (:), and weakly similar residues by *dots* (.). The residues conserved in an alignment of 15 human START domain-containing proteins (32) are indicated in *bold* and the START domains in the proteins are shown by the *underline*.

Previous in-depth analyses have revealed that *P. falciparum* can export hundreds of proteins to the host erythrocyte (9, 10, 12). To determine whether the sequences of the conserved exported *P. falciparum* proteins previously identified by van Ooij *et al.* (11) could reveal insight into their function, we examined them using position-specific iterative-BLAST analysis. This algorithm applies an iterative approach to identify patterns of conservation and then uses these patterns for subsequent searches of the protein database, thereby allowing the identification of weakly conserved regions (27). In the case of PFA0210c this revealed similarity with START domain-containing 7 (StarD7), a phosphatidylcholine transfer protein (28) (Fig. 1) involved in the transfer of phosphatidylcholine to mitochondria (29). Further iterations also identified similarities between PFA0210c and STARD10, a phospholipid transfer protein that can transfer phosphatidylcholine and phosphatidylethanolamine and that belongs to the same family of START domain-containing proteins as StarD7 (30). This finding is consistent with the current annotation of PFA0210c in PlasmoDB version 9.1 (31) as containing a domain belonging to the Bet-v1 family, a superfamily of domains that includes the START domain-containing proteins. The similarity between PFA0210c and StarD7 extends throughout the annotated Bet-v1 domain (residues 149 to 386, *underlined* in Fig. 1), although PFA0210c possesses an additional C-terminal extension of ∼80 amino acid residues.

Further evidence for the inclusion of PFA0210c within the START domain-containing protein family derived from an alignment of human START domain-containing proteins that revealed three absolutely conserved residues and one residue that varied in only one protein (32). Importantly, three of these four residues are also conserved in PFA0210c (Trp-227, Asp-289, and Arg-324; highlighted in *green* in supplemental Fig. S1), whereas the fourth residue, Trp-154 in StarD7 (highlighted in *yellow* in supplemental Fig. S1), is not conserved.

Structure predictions of PFA0210c using pGenTHREADER (17, 33) identified human phosphatidylcholine transfer protein (PDB 1LN1 (34)) (supplemental Fig. S1), STAR-related lipid transfer protein from *Xanthomonas axonopodis* (PDB 3QSZ, Northeast Structural Genomics Consortium), and the human ceramide transport protein (CERT) (PDB 2E3N (35))^4^ as the three proteins with a structure most similar to that predicted for PFA0210c. Although primary sequence identity between PFA0210c and the identified phospholipid transfer proteins is low in each case (14.8, 15.3, and 11.3%, respectively), it is nonetheless significant that all three are lipid transfer proteins. We interpret this as further evidence that PFA0210c may also act as a phospholipid transfer protein.

PFA0210c is conserved among all sequenced *Plasmodium* species (supplemental Fig. S1). Aligning the sequence of PFA0210c with its orthologs from *P. knowlesi*, which infects primates and humans, and *Plasmodium chabaudi*, a rodent malaria parasite, revealed that the sequence comprising the putative START domain contains the highest level of conservation; similarity in this region of the protein is 56% between PFA0210c and PKH_020910 and 48% between PFA0210c and PCHAS_020730. Noticeably less conservation exists in other parts of the protein; similarity over the entire length of the protein is 38% between PFA0210c and PKH_020910 and 33% between PFA0210c and PCHAS_020730. However, despite the low level of similarity in the N-terminal region, all *Plasmodium* orthologs examined contain a putative HT/PE*X*EL motif, suggesting that all are likely to be exported as previously shown experimentally for PFA0210c (11). Collectively, these data suggest that PFA0210c is a conserved, exported phospholipid transfer protein.

##### PFA0210c Is Synthesized during the Asexual Intraerythrocytic Life Cycle and Is Membrane Associated

**FIGURE 2.**
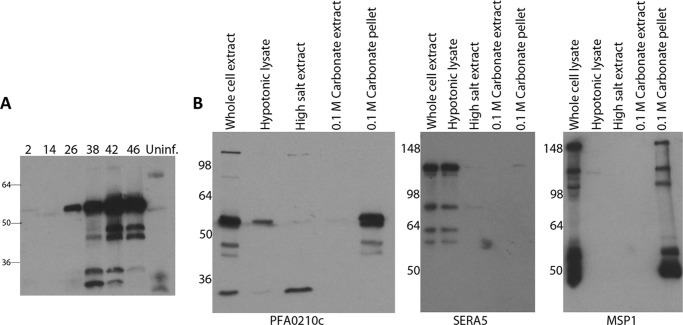
**PFA0210c is expressed in asexual blood-stage parasites as a membrane-associated protein.** *A,* time course of PFA0210c synthesis. Synchronous parasite cultures were analyzed at the indicated time post-invasion by Western blot using anti-PFA0210c mAb NIMP.M11. As a control an extract of uninfected erythrocytes (*Uninf*.) was included. PFA0210c is detectable at very early times post-infection (2 h), is absent during the ring stage (14 h), but is present from the mid-trophozoite stage (26 h) through to the end of the life cycle (46 h). The accumulation of lower molecular weight species recognized by the mAb suggests that the protein is proteolytically processed. *B,* fractionation of parasitized erythrocytes. Soluble, peripherally membrane-associated (high salt or 0.1 m carbonate soluble) and integral membrane or cytoskeletal (0.1 m carbonate insoluble) fractions of parasite extracts were prepared as described under “Experimental Procedures,” then analyzed by Western blot with anti-PFA0210c mAb NIMP.M11. Full-length PFA0210c was detected in the whole cell lysate and the carbonate-insoluble fraction, with a small amount present in the hypotonic lysate. Note that the larger of the lower molecular weight bands detected by the anti-PFA0210 mAb appears to be membrane bound, whereas the smaller of the lower molecular weight bands appears to be soluble in high salt extract, indicating a weaker membrane interaction. As controls, the samples were reprobed with mAb 24C6.1F1 against the soluble PV parasite protein SERA5, as well as mAb X509 specific for the glycosylphosphatidylinositol-linked merozoite surface protein MSP1. Both control proteins were detected in the expected fraction.

Transcriptional studies have indicated that *PFA0210c* mRNA is present during the intraerythrocytic cycle (36–38) and proteomic studies have detected PFA0210c in merozoites (39) and gametocytes (40), indicating that the protein is produced during the asexual intraerythrocytic cycle as well as the sexual stages. Furthermore, a proteomic study detected the *Plasmodium vivax* ortholog PVX081550 in samples taken from malaria patients (41). To determine the profile of PFA0210c expression throughout the asexual intraerythrocytic cycle, extracts of synchronized parasite cultures were prepared at regular intervals and examined by Western blotting using a PFA0210c-specific mAb. This revealed that the protein is detectable very soon (2 h) after invasion, in accord with proteomic data indicating that the protein is present in merozoites (39), and potentially pointing to a role during invasion. The protein was undetectable during the early trophozoite (ring) stage (14 h post-invasion), but by the mid-trophozoite stage (26 h post-invasion), PFA0210c began to accumulate, peaking at the late schizont stage (42–46 h post-invasion) (Fig. 2*A*). This protein expression profile is similar to the transcriptional profile of *PFA0210c* (36, 37), which shows the lowest levels of transcript at late ring stages and a peak of expression during schizogony. Interestingly, the Western blot data showed that, as well as the ∼53-kDa putative full-length PFA0210c gene product, several lower molecular weight products were detectable in parasite extracts, suggesting that the protein may undergo proteolytic processing. To distinguish between the possibilities that the lower molecular weight bands were proteolytic cleavage products or cross-reactive proteins, we performed Western blot analysis on extracts of the transgenic *P. falciparum* strain PfBLD397, which in addition to the endogenous *PFA0210c* gene expresses a second genomic copy of the gene fused to a His_6_ tag, a triple HA tag, and GFP. In this fusion protein, the His_6_-HA_3_-GFP module is inserted between amino acids 117 and 118 of PFA0210c (see supplemental Fig. S2 for a description of the fusion). The gene encoding the fusion protein (called PFA0210c-His_6_-HA_3_-GFP) under control of the 1938 bp of the native promoter region was inserted into the genomic attB site in the 3D7-attB strain, and thus was expected to be transcriptionally regulated similarly to the wild type gene. When extracts of PfBLD397 were probed with the anti-PFA0210c mAb, in addition to the band corresponding to the wild type protein a prominent signal at ∼90 kDa was detected, corresponding to the expected molecular mass of PFA0210c-His_6_-HA_3_-GFP. Importantly, several lower molecular mass bands were detected below the PFA0210c-His_6_-HA_3_-GFP band, in a pattern similar to the pattern detected below the native PFA0210c signal (supplemental Fig. 2*B*). Probing the PfBLD397 extract with anti-HA antibodies revealed the same pattern of bands between 55 and 90 kDa. None of these species were detected in extracts of the parental 3D7-attB parasite line when probed with either antibody, indicating that the bands represent cleavage products of PFA0210c-His_6_-HA_3_-GFP (supplemental Fig. 2*B*).

To determine whether the cleavage of PFA0210c occurs under physiological conditions within the parasite or is instead the result of artifactual cleavage due to release of proteases during extraction of parasite proteins, we mixed recombinant MBP-PFA0210c-His_6_ with schizonts prior to lysis and SDS extraction. Under these conditions, no proteolysis of the recombinant protein was detectable, although the same PFA0210c-derived proteolytic cleavage products as seen in Fig. 2*A* were detected in the parasite extracts (supplemental Fig. 2*C*). It was concluded that limited proteolytic processing of PFA0210c occurs *in vivo* within the parasite.

StarD7 has been shown to interact with membranes (42–44), presumably as a result of its role in transferring phospholipid to membrane structures. To determine whether PFA0210c shares similar characteristics, we next sought to determine whether PFA0210c associates with membranes in the parasitized erythrocyte. Using an extraction protocol that differentiates soluble proteins from those peripherally associated with membranes and integral membrane proteins (see “Experimental Procedures” for details), we found that the lower molecular mass forms of PFA0210c are present as soluble proteins in schizont extracts, whereas the putative full-length form of the protein possesses the characteristics of an integral membrane protein (Fig. 2*B*). These findings are consistent with the predicted function of PFA0210c, and moreover raise the possibility that differential post-translational processing may play a role in regulating its association with membranes.

##### PFA0210c and Its Plasmodium Orthologs Can Transfer Phosphatidylcholine

**FIGURE 3.**
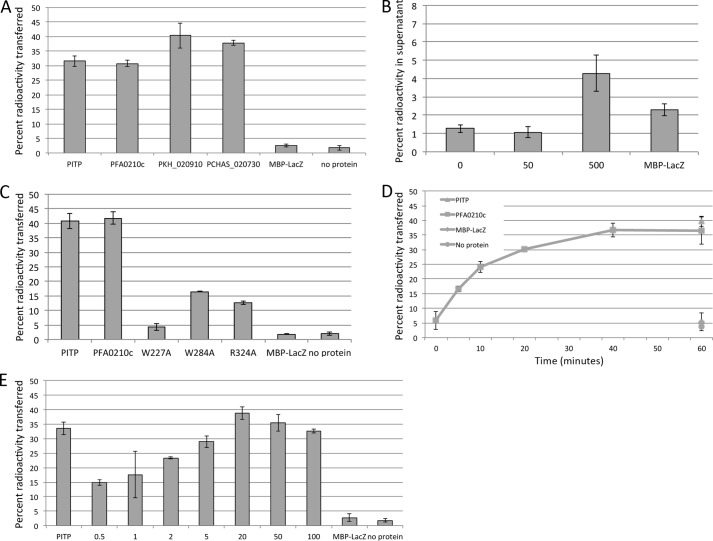
**PFA0210c and its orthologs possess phosphatidylcholine transfer activity.** *A,* phosphatidylcholine transfer activity of PFA0210c and its orthologs, PKH_020910 and PCHAS_020730. Phospholipid transfer was measured by the transfer of radioactive phosphatidylcholine from donor vesicles to acceptor vesicles as described under “Experimental Procedures.” The phosphatidylinositol transfer protein PITP is included as a positive control. The MBP-LacZ fusion and the no protein samples serve as negative controls. *B,* PFA0210c can bind phospholipids directly. A transfer assay was set up as in *A*, except that no donor vesicles were added. At the concentration of protein used in the experiment in *A*, no accumulation of radioactivity in the supernatant is detected, but adding 10-fold more protein results in accumulation of radioactivity in the supernatant. *Numbers below* the axis indicate the concentration of MBP-PFA0210c-His_6_ in μg/ml. *C,* transfer activity of point mutants of PFA0210c. Phosphatidylcholine transfer of PFA0210c carrying a mutation at the indicated position was measured using the same assay as in *panel A*. Note that mutation of the highly conserved residue Arg-324 (see Fig. 1) does not fully inactivate the protein. *D,* time dependence of phosphatidylcholine transfer by PFA0210c. The concentration of PFA0210c used was 5 μg/ml. *E,* dose dependence of PFA0210c-dependent phosphatidylcholine transfer. Numbers below the axis indicate the concentration of MBP-PFA0210c-His_6_ in μg/ml.

To investigate the potential phospholipid transfer activity of PFA0210c, we expressed a portion of the protein, including the putative START domain (residues 144 to 418) as well as the corresponding region from the *Plasmodium knowlesi* (PKH_020910) and *P. chabaudi* (PCHAS_020730) orthologs. All three recombinant proteins were produced as fusions to MBP, and included a C-terminal His_6_ tag. The purified recombinant proteins were then tested for their capacity to transfer phosphatidylcholine using a previously described transfer assay in which donor vesicles consisting of phosphatidylcholine, phosphatidic acid, *N*-lactosylethanolamine, and a radiolabeled phospholipid are mixed with acceptor vesicles containing phosphatidylcholine and phosphatidic acid. After incubation with recombinant protein to allow transfer of phospholipids to occur, the donor and acceptor vesicle populations are separated by agglutination and centrifugation of the donor vesicles. The residual radioactivity in the supernatant, which contains just the acceptor vesicles and the recombinant protein, reflects the amount of phospholipid transferred. As a positive control for these assays we used the well described phospholipid transfer protein PITPα, which can transfer phosphatidylinositol and phosphatidylcholine (45). As shown in Fig. 3*A*, all three recombinant *Plasmodium* orthologs transferred PC at levels similar to that of the control protein PITP. No transfer activity was detected in the absence of acceptor vesicles, indicating that the observed activity reflects transfer to acceptor vesicles and is not the result of association of radiolabeled lipid with the input recombinant protein (Fig. 3*B*). However, when a 10-fold higher level of transfer protein was used in this assay, accumulation of radioactivity could be detected in the supernatant, at significantly higher levels than that accumulated when the same high concentration of MBP-LacZ was added, indicating that the protein can bind directly to radioactive lipids (Fig. 3*B*).

Importantly, substitution of Trp-227, one of the highly conserved START domain residues (Fig. 1), with an Ala residue virtually abolished the transfer activity of recombinant PFA0210c. Substitution with Ala of another highly conserved residue, Arg-324, led to a smaller decrease in activity, as did Ala substitution of Trp-284, a residue that is conserved among the *Plasmodium* orthologs, (Fig. 3*C*) (the corresponding residue in StarD7 is a Tyr residue).

Measurements of the time dependence of the phospholipid transfer reaction showed that the initial rate of transfer was ∼0.34 pmol/min/μg of protein (Fig. 3*D*), which is of the same order as that measured for PITP (not shown). Similarly, we measured the concentration dependence of the reaction. Although this varied somewhat between different batches of recombinant protein, we found that even at concentrations as low as 0.5 μg/ml, significant levels of transfer could readily be detected in a 30-min transfer assay (Fig. 3*E*). Together these results provide evidence that PFA0210c can transfer phosphatidylcholine between phospholipid vesicles and hence act as a *bona fide* phospholipid transfer protein.

##### The Entire START Domain of PFA0210c Is Required for Phospholipid Transfer Activity

**FIGURE 4.**
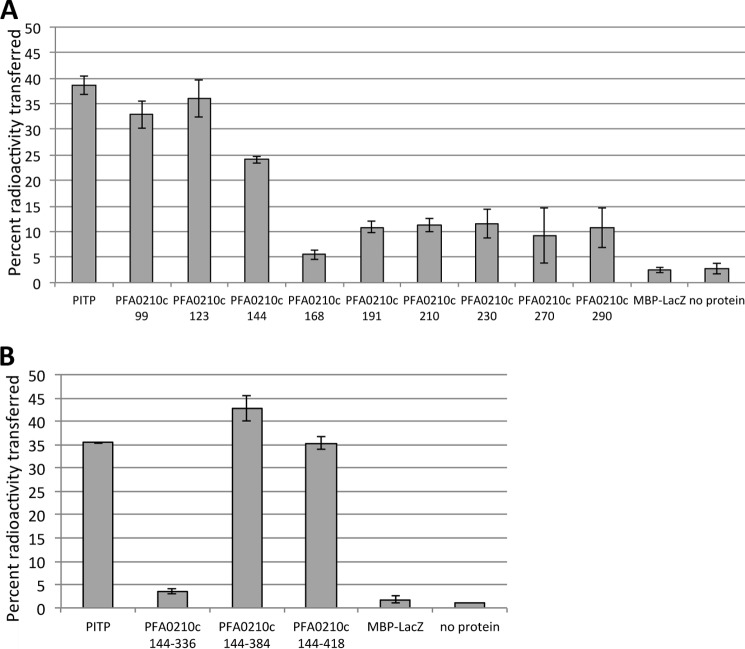
**The phosphatidylcholine transfer activity of PFA0210c resides within the START domain.** *A,* phosphatidylcholine transfer activity of N-terminal truncations. The transfer activity of versions of MBP-PFA0210c-His_6_ containing a portion of PFA0210c beginning at the indicated N-terminal residue were tested using the same transfer assay as described in the legend to Fig. 3*A*. PFA0210cN99, PFA0210cN123, and PFA0210cN144 contain the entire START domain; PFA0210cN168 through PFA0210cN290 lack increasingly large portions of the START domain. *B,* phosphatidylcholine transfer activity of C-terminal truncations. The transfer activity of the MBP-PFA0210c-His_6_ containing the indicated region of PFA0210c was tested. The versions (or constructs) containing residues 144–384 and 144–418 contain the entire START domain.

All orthologs of PFA0210c contain a polypeptide sequence in addition to the annotated START domain, although as observed above this sequence is not highly conserved between the orthologs (supplemental Fig. S1). To explore whether the additional sequence plays a part in phospholipid transfer activity and to determine the minimal domain required for phospholipid transfer, we produced C-terminal and N-terminal truncations of PFA0210c and assessed them using the radioactive phospholipid transfer assay. The N terminus of the protein contains the signal sequence (amino acids 1–24) and the HT motif (amino acids 60–65), which are likely removed during the course of export (46). Similarly, as the region immediately following the HT has been shown to be important for export (14, 15, 47), it is unlikely to have a role in the function of the exported protein. Furthermore, the segment between amino acid residues 65 and 99 is not conserved between PFA0210c and its *Plasmodium* orthologues, making it unlikely that it has a conserved function. For our analysis of the functional domain of PFA0210c, we therefore used recombinant constructs starting at amino acid residue 99. Our assays showed that recombinant proteins containing amino acid residues 99, 123, or 144 (PFA0210cN99, PFA0210cN123, and PFA0210cN144, respectively) as the N-terminal residue can transfer phospholipids, although the transfer activity of PFA0210cN144 was slightly lower than that of PFA0210cN99 and PFA0210N123. When PFA0210c was truncated further, as in the cases of PFA0210cN168 through PFA0210ccN290, no transfer activity was detected. PFA0210cN99, PFA0210cN123, and PFA0210cN144 contain the entire annotated START domain, whereas PFA0210cN168 through PFA0201cN290 lack increasingly large portions of the START domain (Fig. 4*A*). The slightly decreased activity of PFA0210cN144 could be the result of the close proximity of the MBP portion of the fusion protein.

A C-terminal truncation that terminates at residue 384 (PFA0210cC384), the exact end of the annotated START domain, was found to be as active as PFA0210cC418, which terminates at residue 418. In contrast, PFA0210cC336, which only includes the sequence up to residue 336, and thus lacks more than 49 residues of the C-terminal portion of the START domain, was completely inactive in the transfer assay (Fig. 4*B*). We concluded that the minimal phospholipid transfer domain of PFA0210c is defined by amino acids 144–384, showing that the START domain of the protein contains all the structural requirements for transfer activity.

##### PFA0210c Can Transfer Multiple Phospholipids

**FIGURE 5.**
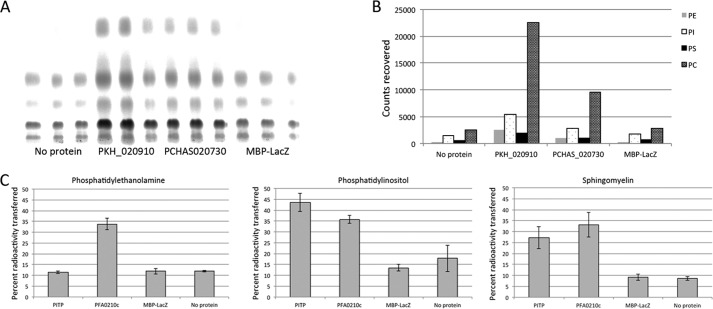
**PFA0210c and its *Plasmodium* orthologs can efficiently transfer several different phospholipids.** *A,* phospholipid transfer from radiolabeled HL60 cells to acceptor vesicles by PKH_020910 and PCHAS_020730. Transfer of phospholipids from the cells to the acceptor vesicles was set up as described under “Experimental Procedures” and the lipids transferred were identified by analytical thin layer chromatography, followed by exposure of the TLC plate to a phosphorimager screen. *B,* quantitation of the phospholipid transfer detected in *panel A*. Background values of the *No protein* sample were used as baseline and were subtracted from the values of the transfer by the *Plasmodium* proteins and the control protein. Quantitation was performed as described under “Experimental Procedures.” *C,* transfer of phosphatidylethanolamine, phosphatidylinositol, and sphingomyelin by PFA0210c was measured using the same transfer assay as in Fig. 3*A*, except that radioactive phosphatidylethanolamine, phosphatidylinositol, or sphingomyelin was incorporated into the donor vesicles. The higher background levels of transfer seen in the negative controls in the case of phosphatidylethanolamine and phosphatidylinositol likely reflect the presence of impurities within the preparations of the radioactive lipids (see “Experimental Procedures”).

Most phospholipid transfer proteins display a profound preference for specific phospholipids, although some can transfer several different phospholipids (49). To test the ability of PFA0210c and the *P. knowlesi* and *P. chabaudi* orthologs to transfer phospholipids other than PC, we initially tested which phospholipids could be transferred by PKH_020910 and PCHAS_020730 in a permeabilized cell assay. In this assay, the test protein is incubated with permeabilized cells that have been labeled with [^14^C]acetate, and therefore contain a wide variety of radiolabeled phospholipids, in the presence of acceptor vesicles. After separation of the cells and the acceptor vesicles, the identity of the phospholipids transferred to the vesicles can be determined by analytical TLC. This revealed that PKH_020910 and PCHAS_020730 can transfer phosphatidylethanolamine, phosphatidylinositol, and phosphatidylserine in addition to phosphatidylcholine (Fig. 5, *A* and *B*). Minimal transfer activity was detected in the presence of MBP-LacZ or in the absence of protein. To verify that PFA0210c can also transfer phosphatidylethanolamine and phosphatidylinositol, we produced donor vesicles containing purified ^14^C-labeled phosphatidylethanolamine or phosphatidylinositol. PFA0210c was able to transfer both of these phospholipids (Fig. 5*C*), whereas the control protein PITP was able to transfer only PI, as expected (45). In addition, PFA0210c was also able to transfer sphingomyelin, similar to PITP, which has previously been shown to transfer sphingomyelin (50). This result strongly suggests that PFA0210c and its *Plasmodium* orthologs have the capacity to transfer multiple phospholipids.

To estimate the relative affinities of PFA0210c and its orthologs for different phospholipids, we compared the ratios of radioactive phospholipids in both the HL60 cell extracts and acceptor vesicles used in Fig. 5, *A* and *B*. To do this, we arbitrarily used levels of transferred phosphatidylinositol as a reference, assigning a value of 1 to that value and then determining the relative levels of the other radiolabeled phospholipids in the HL60 cell extract compared with those transferred to acceptor vesicles. Comparison of these two ratios for each phospholipid then allowed for a comparison of the relative efficiency of transfer. The analysis revealed that the relative level of phosphatidylcholine in the cells and vesicles was very similar to that of phosphatidylinositol, and that the level of phosphatidylserine was only slightly decreased in the vesicles (Table 1). However, the level of phosphatidylethanolamine in the vesicles was reduced about 3-fold compared with the level of phosphatidylethanolamine in the HL60 cell extract, indicating that PFA0210c orthologues have a relative affinity for phospholipids as follows: PI = PC > PS ≫ PE. As phosphatidylserine is found exclusively in the inner leaflet of the cell plasma membrane, we judged that the test proteins must have had access to the cytosol of HL60 cells during the course of the assay. The lower levels of transfer of phosphatidylethanolamine therefore did not result from a lack of access of the transfer protein to the phospholipid. Levels of other phospholipids in the HL60 cell extract were too low to be used for the determination of the relative transfer efficiency (data not shown).

**TABLE 1:** **Relative phospholipid content of HL60 cells and vesicles after incubation with PKH_020910 and PCHAS_020730. Phosphatidylinositol was used as standard; values reflect the ratio of the indicated phospholipid to phosphotidylinositol. Data were taken from the experiment shown in Fig. 5*A***

	HL60	PKH_020910	HL60/PKH_020910	PCHAS_020730	HL60/PCHAS_020730
Phosphatidylinositol	1	1	1	1	1
Phosphatidylcholine	5.11	4.92	1.04	4.91	1.04
Phosphatidylethanolamine	1.51	0.55	2.75	0.56	2.70
Phsophatidylserine	0.41	0.35	1.17	0.37	1.11

##### Localization of PFA0210c-GFP

**FIGURE 6.**
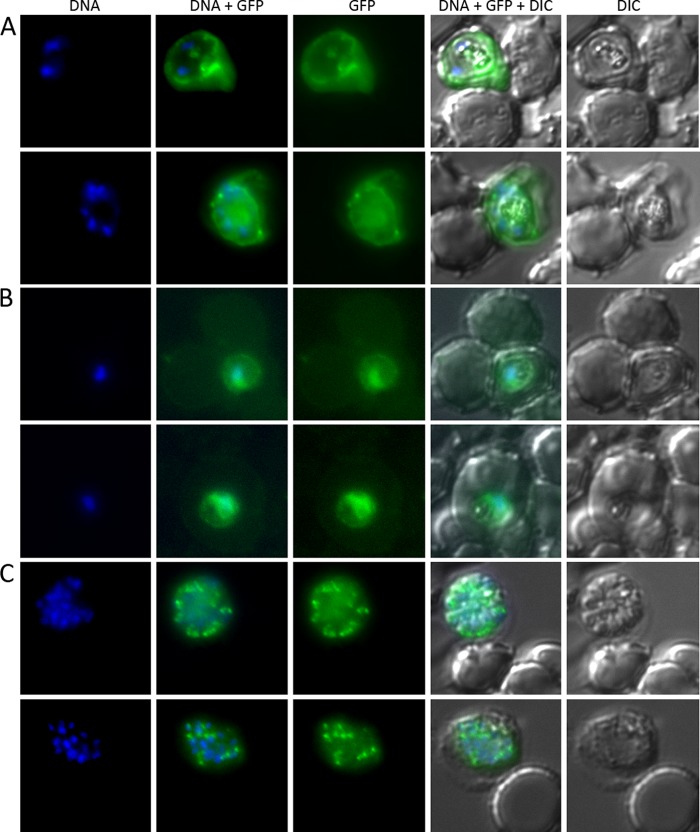
**PFA0210c is an exported *P. falciparum* protein.** *A,* the PFA0210c-His_6_-HA_3_-GFP fusion can be exported into the erythrocyte cytosol. In *P. falciparum* strain PfBLD390, which produces the same GFP fusion as PfBLD397, but where expression is controlled by the stronger calmodulin promoter, the GFP fusion is detected in the erythrocyte cytosol, indicating export beyond the PVM boundary. *B* and *C,* PfBLD397, expressing a fusion of PFA0210c to GFP controlled by its native promoter (see supplemental Fig. S2 for description of the fusion), was imaged at the early trophozoite stage (28 h after invasion; panels in *B*) and the schizont stage (46 h after invasion; panels in *C*). In the trophozoite stage, the protein is present in the characteristic circular pattern of the PV. In the schizont stage, the protein is recruited to organelles within the parasite.

To obtain insight into the possible role of PFA0210c in the parasite, we sought to determine its subcellular localization. Our previous study that reported export of PFA0210c used a C-terminal fusion to GFP expressed under control of the strong *P. falciparum* calmodulin promoter (11). To confirm that the PFA0210c-His_6_-HA_3_-GFP fusion can also be exported, we produced a new construct designed to express PFA0210c-His_6_-HA_3_-GFP under control of the calmodulin promoter and introduced this into *P. falciparum*. GFP fluorescence was readily detected in the cytosol of the resulting parasite line (Fig. 6*A*), confirming that PFA0210c is an exported parasite protein. Examination of PFA0210c-His_6_-HA_3_-GFP expressed in synchronized PfBLD397 parasites at ∼28 h post-invasion (early trophozoite stage) revealed a much lower level of fluorescence (likely due to its expression from the native PFA0210c promoter in these parasites) and the erythrocyte cytosol signal was not obvious, likely due to fluorescence quenching by host cell hemoglobin. However, the visible GFP signal was still concentrated in a circular pattern indicative of localization in the PV (Fig. 6*B*), again confirming export beyond the parasite plasma membrane. Interestingly, examination of mature PfBLD397 schizonts revealed the fusion protein to be localized primarily in small compartments surrounding the edge of the parasite, suggesting that it could be transferred to an apical organelle in mature developmental stages (Fig. 6*C*). This is consistent with the finding that PFA0210c can be detected in merozoites (39).

To finally determine whether native, unmodified PFA0210c is exported during the stage of the life cycle shown in Fig. 6*A*, we treated *P. falciparum*-infected erythrocytes with streptolysin O, which permeabilizes the erythrocyte membrane but not the PVM and so selectively releases proteins present in the erythrocyte cytosol (23). We found that upon streptolysin O treatment, over half of the PFA0210c was released into the supernatant (supplemental Fig. S3). In contrast, a much smaller fraction of the PV resident protein PfPV1 (51) was released (supplemental Fig. S3), indicating that the PVM remained largely intact during streptolysin O treatment. Together, these experiments show that PFA0210c is present in the PV and is also exported into the erythrocyte during the trophozoite stage and that it is recruited into a parasite organelle during schizogony.

## DISCUSSION

Previous identification by us and others of the full complement of *P. falciparum* proteins that are exported to the host erythrocyte has revealed that interactions between the parasite and its host cell are much more complex than previously thought. However, in only a very few cases is the function of exported proteins known. Here we have focused on the biochemical function of PFA0210c, an exported protein that is conserved among *Plasmodium* species and therefore likely performs a conserved role. Bioinformatic analysis showed that this protein is a member of the START domain-containing family. This was confirmed experimentally by functional assays that showed that PFA0210c can transfer phosphatidylcholine, phosphatidylethanolamine, phosphatidylinositol, and sphingomyelin between phospholipid vesicles. This action appears to be rapid and is mediated with an efficiency quantitatively similar to that of the well described phosphatidylinositol transfer protein PITP. No binding of fatty acids by PFA0210c was detected in a fatty acid-binding assay (not shown), indicating that the activity of PFA0210c is specific for phospholipids. We conclude that PFA0210c is a *bona fide* phospholipid transfer protein with a broad specificity for phospholipid transfer. Orthologs from *P. knowlesi* and *P. chabaudi* displayed the same phospholipid transfer activity, and therefore phospholipid transfer is likely a conserved requirement for all *Plasmodium* parasites. As all known PFA0210c orthologs contain an HT/PE*X*EL export motif, the phospholipid transfer activity mediated by these proteins is likely to be an important function in the infected erythrocyte. Transgenic expression in *P. falciparum* of PFA0210c and streptolysin O-mediated permeabilization experiments confirmed that PFA0210c is an exported protein.

The precise role of PFA0210c and its orthologs remains to be determined. The non-parasitized mature erythrocyte is devoid of internal membranes, so the membrane-bound compartments in the infected erythrocytes, the tubovesicular network, the Maurer clefts, and the PVM must be made *de novo*. As the surface area of the parasitized erythrocyte does not change much as the parasite matures (52), the parasite itself is the likely source of these membranes and the phospholipids that form them. There is no evidence that these compartments are contiguous with the plasma membrane of the parasite, necessitating a transport system for the phospholipids. As there is no evidence for vesicular transport directly from the parasite plasma membrane into the PV, we propose that PFA0210c may deliver the phospholipids required for the expansion of the PV. The tubovesicular network appears to be contiguous with the PVM (53, 54), and although the attachment of the Maurer clefts to the PVM remains unclear (55–57), as an exported protein, PFA0210c could provide the building blocks for all the internal organelles. This hypothesis requires rigorous genetic and biochemical evaluation, a focus of ongoing work. It should be noted that the obligate intracellular parasite *Chlamydia trachomatis* appears to use a similar strategy to obtain sphingomyelin for the expansion of the membrane-bound compartment (inclusion) in which it resides by recruiting the host ceramide transfer protein to the inclusion to obtain ceramide synthesized by the host cell (48, 58). Attempts to delete the *PKH*_*020910* gene in *P. knowlesi* have been unsuccessful,^5^ providing preliminary evidence that the protein is essential. As the sequence outside of the START domain is not well conserved, the function of these parts of the protein also requires further investigation.

This study has shown that in depth bioinformatic analysis supplemented by biochemical analysis can establish biochemical functions of exported *Plasmodium* proteins, in this case phospholipid transfer. Study of additional exported proteins in this manner may begin to provide a more complete picture of the interaction of the malaria pathogen with its host erythrocyte.

## Footnotes

4 C. van Ooij, C. Withers-Martinez, and M. J. Blackman, unpublished data.

5 C. van Ooij, R. Moon, and M. J. Blackman, unpublished data.

## Footnotes

3 The abbreviations used are:

PV: parasitophorous vacuole
PVM: PV membrane
START: steroidogenic acute regulatory protein-related lipid transfer
PE: phosphatidylethanolamine
PI: phosphatidylinositol
PC: phosphatidylcholine
MBP: maltose-binding protein
PDB: Protein Data Bank
PITP: rat phosphatidylinositol transfer protein α
NTA: nickel-nitrilotriacetic acid.
